# Assessing the Clinical Value of Performing CT Scan before Rhinoplasty Surgery

**DOI:** 10.1155/2020/5929754

**Published:** 2020-12-18

**Authors:** Hesam Jahandideh, Mojtaba Maleki Delarestaghi, Delaram Jan, Ayda Sanaei

**Affiliations:** ^1^ENT and Head & Neck Research Center, The Five Senses Institute, Iran University of Medical Science, Tehran, Iran; ^2^Department of Otolaryngology Head and Neck Surgery, Firoozgar Hospital, Iran University of Medical Science, Tehran, Iran; ^3^ENT and Head and Neck Research Center and Department, Five Senses Health Research Institute, Hazrat Rasoul Akram Hospital, Tehran, Iran

## Abstract

**Introduction:**

The endonasal mucosal or anatomic pathologies could lead to poor functional results and dissatisfaction after rhinoplasty. Although computed tomography (CT) scan has become an integral part of the diagnostic paradigm for patients with pathologies of the paranasal sinuses, the use of CT scan for preoperative evaluation of patients seeking rhinoplasty is up for debate. Our aim in this study was to compare the efficacy of CT scan in diagnosing nasal pathologies with other evaluating tools in patients undergoing rhinoplasty.

**Design:**

In this randomized controlled trial study, 74 consecutive patients seeking cosmetic rhinoplasty referred to otorhinolaryngology clinic were randomly assigned into three groups based on the perioperative evaluation method: the CT group, the nasal endoscopy group, and the control group (anterior rhinoscopy only). Surgical planning was made according to perioperative findings, and the identified endonasal pathologies were corrected during the surgery. The functional and aesthetic outcomes of the rhinoplasty were assessed by Nasal Obstruction Symptom Evaluation (NOSE), Rhinoplasty Outcome Evaluation (ROE), and the Visual Analogue Scale (VAS) tools before surgery and at 12-month follow-up.

**Results:**

All outcome measures improved significantly in either group toward one year after rhinoplasty (all with *p* value <0.05). Subjects in the CT group demonstrated greater improvement in the NOSE, VAS, and ROE compared to other two groups (NOSE: *p* value = 0.17; VAS: *p* value = 0.024; ROE: *p* value = 0.042).

**Conclusions:**

According to our study, perioperative CT is associated with greater patients' satisfaction and quality of life after rhinoplasty compared to either nasal endoscopy or anterior rhinoscopy. A preoperative CT scan may improve the outcomes of rhinoplasty.

## 1. Introduction

Patients' satisfaction after rhinoplasty is one of the most important parameters of success and depends on good functional and aesthetic results [1]. According to previous studies, the primary reason for revision rhinoplasty is nasal tip asymmetry followed by breathing difficulties or nasal obstruction [2]. Therefore, the endonasal mucosal or anatomic pathologies which could lead to poor functional results and dissatisfaction should not be neglected when planning for surgery [3]. A thorough perioperative assessment of patients can lead to proper patient selection, planning, and better functional and aesthetic outcomes [2].

Several evaluation methods are used for perioperative assessment including acoustic rhinometry, rhinoscopy, nasal endoscopy, magnetic resonance imaging (MRI), and computed tomography (CT) [4]. Anterior rhinoscopy is often the first diagnostic procedure to evaluate obstructive nasal pathologies although it is unable to assess the posterior nasal cavity and the middle meatus, properly. Due to this shortcoming, nasal endoscopy is considered to be the gold standard for diagnosing and grading of a deviated septum and turbinate hypertrophy. However, the CT scan is the method of choice for the evaluation of paranasal anatomy and inflammatory paranasal sinus pathologies [5, 6]. CT imaging can provide surgeons with vital information about the entire septum, as well as anatomical structures of the nasal valve region, nasal walls, and nasal tip [7, 8].

CT scan is an essential part of the diagnostic paradigm for patients with clinical conditions involving paranasal sinuses, considering its vital and complete provided information; however, the use of routine CT for preoperative evaluation of patients seeking rhinoplasty is up for debate [9]. In this study, our goal was to evaluate the role of CT scan in the identification of nasal pathologies in patients undergoing rhinoplasty and its effect on surgical outcomes. Furthermore, we compared the results with other evaluating tools, i.e. nasal endoscopy and anterior rhinoscopy.

## 2. Material and Methods

### 2.1. Study Design and Setting

This randomized controlled trial study was carried out with 74 consecutive patients seeking primary cosmetic rhinoplasty, referring to our otorhinolaryngology clinic from 2017 to 2019. Our Institutional Review Board endorsed this study. The study was approved by the university research ethics committee. The research was conducted according to the Helsinki Declaration, and informed written consent was obtained from all participants.

### 2.2. Participant

Subjects were randomly assigned into 3 treatment groups using randomly generated treatment allocations: the CT group (*n* = 26), the nasal endoscopy group (*n* = 23), and the control group (*n* = 25). The physician who evaluated the outcome measures and the person responsible for data analysis were blinded to the group allocation.

### 2.3. Methods

In either group, anterior rhinoscopy was performed before surgery. In the CT group, patients underwent paranasal sinus CT and coronal images with the sequential techniques were acquired. These images were evaluated by an experienced otorhinolaryngologist to identify the endonasal mucosal or anatomic pathologies. Internal nasal valve angle was measured using PACS software, according to bloom et al.'s study [10]. In the nasal endoscopy group, endoscopy was performed in the examination room before surgery. The nasal endoscopy was performed in a sitting position with a 0° rigid endoscope after topical decongestion by application of tetracaine and epinephrine for 20 minutes. The tip of our 4 mm diameter rigid endoscope was inserted through the nostril, and when the internal nasal valve was visible, images were captured for measuring the area. Complete evaluation of septum and nasal cavity was performed, and findings were recorded. In the control group, perioperative evaluation only included anterior rhinoscopy. Then, patients underwent open septorhinoplasty.

All surgeries were performed by a single surgeon. The surgical approach was planned, some tailored changes were made during the course of surgery according to preoperation findings, including high septal deviation correction, graft implantation (e.g. spreader grafts), resection of concha bullosa, and turbinoplasty techniques. Cases of mild nasal polyposis received a course of medical treatment before surgery. For patients with narrow nose syndrome and patients with a higher risk for internal valve insufficiency (which was evaluated by CT scan and endoscopy), a thick spreader graft was used on the insufficient side [11]. For cases of hyperaeration of maxillary sinus, external osteotomy was performed with great care to avoid complications. Cases who required endoscopic intervention were managed simultaneously.

All participants received similar postoperative care. All patients were discharged within 6 hours after surgery. Nasal packing and external splint were removed on the third and seventh day after the operation, respectively. All subjects returned to the otorhinolaryngology clinic on the 3rd, 7th, 14th, and 28th day and the second, sixth, and twelfth months after surgery for follow-up. None of the patients developed major complications during the follow-ups. There were varying degrees of ecchymosis and edema in all treatment groups, and all of which improved using compression and medical treatment.

### 2.4. Data Recording and Analysis

The outcome of rhinoplasty and patients' symptoms were assessed by Nasal Obstruction Symptom Evaluation Scale (NOSE), Rhinoplasty Outcome Evaluation (ROE), and the Visual Analogue Scale (VAS) to address both functional and cosmetic concerns. All outcome measures were assessed before surgery and at 12-month follow-up. NOSE is a 5-item scoring system used to assess patients' symptoms and quality of life within the past month. Each item is scored on a 5-point Likert scale from 0 (not a problem) to 4 (severe problems), when analyzing, the data multiples by 5. The total score ranges from 0 (no obstructive nasal problem) to 100 (severe problems) [12, 13]. The ROE questionnaire has six questions, two for each factor considered key in patient satisfaction after rhinoplasty (physical, emotional, and social). Each question has five answer options, graded from zero to four with a total score of zero to 24. The final score was divided by 24 and multiplied by 100. The higher the score, the greater is the patient's satisfaction with the nose surgery [14]. Breathing difficulty was also measured using a 10 cm VAS (0 = no problem and 10 = severe problem), and patients were asked to point the place on the VAS ruler representing their breathing problem.

The outcome measure was analyzed using IBM® SPSS® Statistics V 24.0. The results for quantitative and qualitative variables were expressed as mean ± standard deviation and percentage, respectively. The significant threshold was considered to be less than 0.05.

## 3. Results

Participants' demographics are demonstrated in Table 1. No significant difference was observed between study groups regarding sex and age (*p* value = 0.110 and 0.676, respectively).

**Figure 1:**
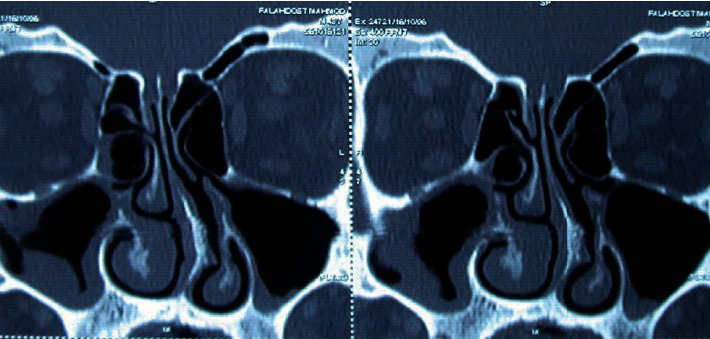
Bilateral mucosal thickening in maxillary sinuses, more severe at the right side.

**Figure 2:**
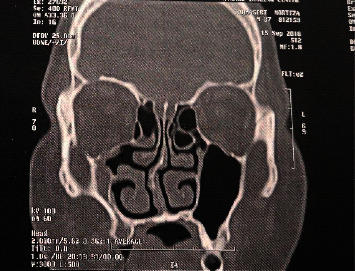
Decreased volume of right maxillary sinus associated with complete opacification in favor of silent sinus syndrome.

Of subjects in the CT group, concha bullosa was observed in 57.69% of cases, varying degrees of septal deviation in 53.84, considerable high deviated septum in 38.46%, nasal valve insufficiency in 34.6%, mucosal thickening in 23.07%, hyperaeration of sinuses in 11.5%, uncinate bullosa in 3.8%, silent sinus syndrome in 3.8%, and visible tooth-root through the maxillary sinus in 3.8%. Among them, two cases of maxillary sinusitis and ostiomeatal complex obstruction (Figure 1) and one case of silent sinus syndrome were managed by endoscopic antrostomy during the same procedure (Figure 2).

The incidence of different degrees of septal deviation, high deviated septum, nasal valve insufficiency, and mucosal thickening was 43.47%, 21.73%, 26.08%, and 21.7%, respectively, in the nasal endoscopy group. Mild nasal polyposis was observed in 2 and 1 cases when evaluating with CT and endoscopy, respectively (Table 2).

All three groups were assessed as baseline. No significant difference was observed between our three treatment groups regarding baseline measures (NOSE: *F* = 0.254, *p* value = 0.776; ROE: *F* = 1.07, *p* value = 0.347; NOSE: *F* = 0.669, *p* value = 0.515).

The course of outcome measures from baseline to 12-month follow-up is demonstrated in Table 3. As it is shown, outcome measures improved significantly in either group 12 months after surgery (all with *p* value <0.05). Regarding NOSE score, the 12-month score was 2.11 ± 3.78 in the CT group, 5.65 ± 5.89 in the nasal endoscopy group, and 5.40 ± 4.54 in the control group (*p* value <0.001, *p* value = .001, and *p* value = 0.003), respectively, compared to baseline values. The course of NOSE was significantly different between our three groups (*F* = 4.303, *p* value = 0.017). The ROE score improved toward the 12-month evaluation reaching to 90.22 ± 6.65 in the CT group (*p* value <0.001), 84.96 ± 9.95 in the nasal endoscopy group (*p* value <0.001), and 83.99 ± 8.89 in the control group (*p* value <0.001). In this group, the difference between the three groups was also significant (*p* value = 0.024). In either group, subjects reported an improved breathing based on VAS reaching to 0.34 ± 0.71 in the CT group (*p* value <0.001), 0.56 ± 1.11 in the nasal endoscopy group (*p* value = 0.007), and 1.04 ± 1.07 in the control group (*p* value = 0.001). The changes in VAS was significantly different between the three treatment groups (*F* = 3.325, *p* value = 0.042).

## 4. Discussion

The primary focus of rhinoplasty in most cases is the improvement of the nasal shape. However, the concomitant pathologies of the nasal cavity are not to be disregarded as they may cause unresolved breathing and sinonasal problems and, thus, dissatisfaction after rhinoplasty [2]. A precise preoperative assessment for accurate determination of endonasal mucosal and structural pathologies is essential to ensure proper planning in primary rhinoplasty [6].

Several authors have reported CT scan as an ideal preoperative tool for facial surgical planning [15]. According to Setzen et al. [9], CT imaging is a key aspect of surgical planning and management of adult patients with sinonasal and skull base pathologies. The application of preoperative CT in septoplasty was described in the literature. Günbey et al. [5] recommended performing preseptoplasty CT evaluation in patients with obstructive middle turbinate hypertrophy, severe anterior septal deviation, nasal polyp, and chronic sinusitis. Karatas et al. [16] reported that preoperative CT can be helpful in patients with nasal obstruction undergoing septoplasty to recognize pathologies that cannot be found on physical examination, allowing further surgical interventions.

Despite the vital contribution of preoperative CT scanning for paranasal sinuses surgical planning, little could be found in the literature concerning preoperative CT before rhinoplasty itself. While a preoperative CT scan for septoplasty has been of debate, we think it should be reconsidered to evaluate the use of preoperative CT evaluation for patients seeking cosmetic rhinoplasty. Due to CT scan limitations, including radiation exposure and increased cost [7, 15, 17], there is an argument on whether preoperative routine CT is truly necessary for these patients. On the other hand, the high prevalence of functional sinonasal symptoms and pathologies in patients seeking cosmetic rhinoplasty and their effects on surgical outcomes requires further studies to determine the impact of preoperative CT in these patients [2].

**Figure 3:**
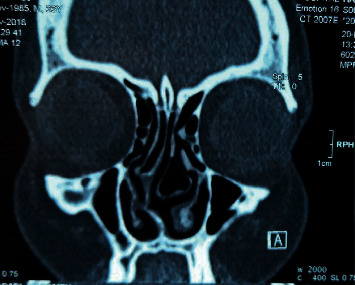
Nasal septal deviation to the right with nasal septal spur. Left concha bullosa causing narrowing of middle meatus.

**Figure 4:**
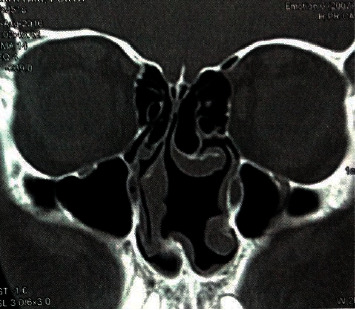
Severe nasal septal deviation to the right side causing narrowing of right middle meatus associated with bilateral concha bullosa.

Based on the findings of the present study, the CT evaluation was associated with an identification of considerable cases of the endonasal pathologies. The most frequent finding in CT images was concha bullosa (57.6%), this pathological finding could not be properly assessed using nasal endoscopy or anterior rhinoscopy (Figure 3). The septal deviation was detected in a higher percentage (total: 53.48%/high SD: 38.46%) of patients in the CT scan group (Figure 4). Other studies have reported a prevalence of concha bullosa and septal deviation of 67.3% and 49.5% [6].

In a retrospective study of 672 CT scans by Yigit et al., concha bullosa ratio in patients with deviated septum was 45.3%, compared to 18.95% in patients without a deviated septum. Also, the presence of unilateral concha bullosa was significantly associated with a higher septal deviation angle [18]. Proper treatment of disorders caused by concha bullosa requires an accurate preoperative diagnosis of the disorder and precise surgical planning.

The prevalence of sinus disease was also higher in the CT images (23.07% compared to 21.7% in the nasal endoscopy group and 8% in the control group). Several other deformities of the nasal cavity were not diagnosed during rhinoscopy and endoscopic evaluation (e.g., hyperaeration, silent sinus syndrome, and protrusion of tooth-root into the maxillary sinus).

According to Günbey et al.'s study [5], nasal endoscopy has low sensitivity and specificity for concha bullosa, mucocele, and CRS found by CT particularly among patients with moderate to severe septal deviation. The accurate identification of endonasal pathologies may lead to some additional surgical changes, including concha bullosa resection and functional endoscopic sinus surgery (FESS) (in the event of concomitant CRS) [5, 18]. Diagnosing more cases of anatomical and mucosal disorders and performing appropriate treatments improve nasal function and therefore patient's satisfaction.

In our study, all subjective outcome measures (i.e., NOSE, ROE, and breathing difficulty based on VAS) improved significantly in either group during 12 months after rhinoplasty and significantly greater changes in NOSE, VAS, and ROE scores were observed in subjects receiving preoperative CT.

Few studies were found in the literature concerning this subject. According to Graviero et al. [4], CT with three-dimensional (3D) reconstruction has proven to be a superior preoperative tool compared to the nasal endoscopy and anterior rhinoscopy. 3D CT scanning could improve the outcomes of rhinoplasty by assessment of the nasal valve, the alar and lateral cartilages, interdomal distance, loss of bone-cartilaginous substance, etc. which are vital parameters for the preoperative planning of rhinoplasty [19].

Other studies suggested that CT scan should be performed in the setting of nasal obstruction without a clear endoscopic diagnosis or when chronic obstructive or inflammatory pathologies are suspected with nasal endoscopy [20, 21]. Tao et al. [22] reported 28 unsuccessful septoplasties, among which the CT evaluation found 6 undiagnosed upper or posterior nasal septal deviation and 12 cases of neglected paraseptal structural deformities and chronic sinus diseases. They concluded that preoperative CT should be performed to detect concomitant nasal disorders.

Suh et al. [23] reported that reconstructed CT scanning is associated with more accurate results and a higher reproducibility compared to an endoscopic evaluation in the measurement of the internal nasal valve (INV) angle. The proper preoperative estimation of the INV and its angle has vital implications when selecting surgical techniques to prevent nasal obstruction after rhinoplasty [10]. Miman et al. [24] also recommended that INV should be examined objectively before any surgery for adequate surgical approach selection. Bakker et al. [25] stated that assessment of anatomical structures involved in nasal passage by measuring its cross-sectional area, using CT scan, can help to determine the most adequate surgical plan to reach maximum functional improvement.

Orhan et al. [26] reported that the preoperative CT is useful to evaluate hypertrophy of inferior turbinate and can aid to decide the surgical technique to fix turbinate. Inferior turbinates are erectile organs that play an important role in nasal breathing, and one of the main reasons for nasal obstruction after septoplasty operations is the inadequate treatment of inferior turbinate dysfunction.

The findings of the present study were in line with the abovementioned studies underlying the importance of preoperative CT for detecting endonasal deformities and their impact on surgical outcomes.

Khojastepour et al. [6] evaluated the incidences of nasal variation in patients seeking rhinoplasty using cone-beam computed tomography (CBCT), a CT system with lower radiation exposure, lower cost, and fewer metal artifacts. They reported a high prevalence of ostiomeatal complex variations and mucosal thickening among patients seeking rhinoplasty and found comparable results between CBCT and regular multislice CT. The prevalence of concha bullosa and deviated septum was 67.3% and 49.5%, respectively, in this study and correlated to the present study (57.69% and 53.84%, respectively). Similar findings were observed in the study of Shokri et al. [27] on the prevalence of anatomical variations of the nasal cavity on CBCT. In Schell et al.'s study [28], dual-source CT was used to evaluate paranasal sinus and facial skull with comparable results to conventional CT. They suggested that with the proposed dual-source mode, good diagnostic image quality with lower radiation exposure could be achieved. These advancements and alteration in CT scanning protocols could further expand the use of preoperative CT in patients seeking rhinoplasty.

In summary, tough the identification of endonasal deformities could be feasible with different evaluation tools, CT scan seems to be a superior method with better subjective outcomes after rhinoplasty compared to anterior rhinoscopy and nasal endoscopy.

Despite the strength of the present study, which was one of the few in the literature to assess the impact of preoperative CT, it has some limitations that should be addressed for further studies. The main limitation was the limited population. We assessed the outcome of the rhinoplasty only through subjective self-reported measures. Despite the historical emphasis on subjective measures, objective measures are gaining importance in both research and clinical setting [29].

## 5. Conclusions

According to our study, a higher prevalence of endonasal pathologies was observed using preoperative CT and it was associated with greater patients' satisfaction and quality of life after rhinoplasty compared to either NE or anterior rhinoscopy. The preoperative CT scan could improve the functional and cosmetic results of rhinoplasty.

## Figures and Tables

**Table 1:** Participants demographics.

Characteristic	CT	Endoscopy	Control	*p* value
Number	26	23	25	
Sex, *n* (%)				
Female	19 (73.1)	15 (65.2)	17 (68)	0.110
Male	7 (26.9)	8 (34.8)	8 (32)	
Age (*year*), *mean* *±* *std*. *deviation*	30.88 ± 7.07	29.47 ± 5.50	29.24 ± 7.01	0.676

**Table 2:** The prevalence of endonasal pathologies in each group.

Characteristic (%)	CT	Endoscopy	Control
Concha bullosa	57.69	—	—
Nasal septum deviation	53.84	43.47	48
*/High septal deviation*	/38.46	/21.73	—
Mucosal thickening	23.07	21.7	8
Nasal polyps, *n*	7.7	4.3^*∗*^	—
Hyperaeration	11.5	—	—
Nasal valve insufficiency	34.6	26.08	—
Uncinate bullosa	3.8	—	—
Maxillary atelectasis	3.8	—	—
Tooth-root through the maxillary sinus	3.8	—	—

^*∗*^2 cases were excluded in this group due to severe polyposis.

**Table 3:** Outcome measure changes during study.

Characteristic	Preoperation	Postoperation	*p* value^a^	*F* value^b^	*p* value^b^
NOSE, mean ± std. deviation					
CT	10.19 ± 9.74	2.11 ± 3.78	<0.001	4.303	0.017
Endoscopy	10.86 ± 10.07	5.65 ± 5.89	0.001		
Control	9 ± 7.77	5.40 ± 4.54	0.003		

ROE, mean ± std. deviation					
CT	42.62 ± 9.81	90.22 ± 6.65	<0.001	3.919	0.024
Endoscopy	37.86 ± 10.64	84.96 ± 9.95	<0.001		
Control	40.00 ± 13.39	83.99 ± 8.89	<0.001		

VAS, mean ± std. deviation					
CT	2.30 ± 2.01	0.34 ± .71	<0.001	3.325	0.042
Endoscopy	1.65 ± 1.96	0.56 ± 1.11	0.007		
Control	1.96 ± 1.97	1.04 ± 1.07	0.001		

^a^Paired samples test, ^b^ANOVA. NOSE: nasal obstruction symptom evaluation scale; ROE: rhinoplasty outcome evaluation; VAS: visual analogue scale.

## Data Availability

The data used to support the findings of this study are included in the article.
